# Global Polio Eradication – Way Ahead

**DOI:** 10.1007/s12098-017-2586-8

**Published:** 2018-01-05

**Authors:** Sunil Bahl, Pankaj Bhatnagar, Roland W Sutter, Sigrun Roesel, Michel Zaffran

**Affiliations:** 1 World Health Organization - Regional Office for South-East Asia, New Delhi, India; 2 National Polio Surveillance Project, World Health Organization, New Delhi, India; 3 World Health Organization, Geneva, Switzerland

**Keywords:** Poliomyelitis, Eradication, Oral poliovirus vaccine, Surveillance, Certification

## Abstract

In 1988, the World Health Assembly resolved to eradicate poliomyelitis by the year 2000. Although substantial progress was achieved by 2000, global polio eradication proved elusive. In India, the goal was accomplished in 2011, and the entire South-East Asia Region was certified as polio-free in 2014. The year 2016 marks the lowest wild poliovirus type 1 case count ever, the lowest number of polio-endemic countries (Afghanistan, Nigeria and Pakistan), the maintenance of wild poliovirus type 2 eradication, and the continued absence of wild poliovirus type 3 detection since 2012. The year also marks the Global Polio Eradication Initiative (GPEI) moving into the post-cessation of Sabin type 2, after the effort of globally synchronized withdrawal of Sabin type 2 poliovirus in April 2016. Sustained efforts will be needed to ensure polio eradication is accomplished, to overcome the access and security issues, and continue to improve the quality and reach of field operations. After that, surveillance (the “eyes and ears”) will move further to the center stage. Sensitive surveillance will monitor the withdrawal of all Sabin polioviruses, and with facility containment, constitute the cornerstones for eventual global certification of wild poliovirus eradication. An emergency response capacity is essential to institute timely control measures should polio still re-emerge. Simultaneously, the public health community needs to determine whether and how to apply the polio-funded infrastructure to other priorities (after the GPEI funding has stopped). Eradication is the primary goal, but securing eradication will require continued efforts, dedicated resources, and a firm commitment by the global public health community.

## Background

**Fig. 1:**
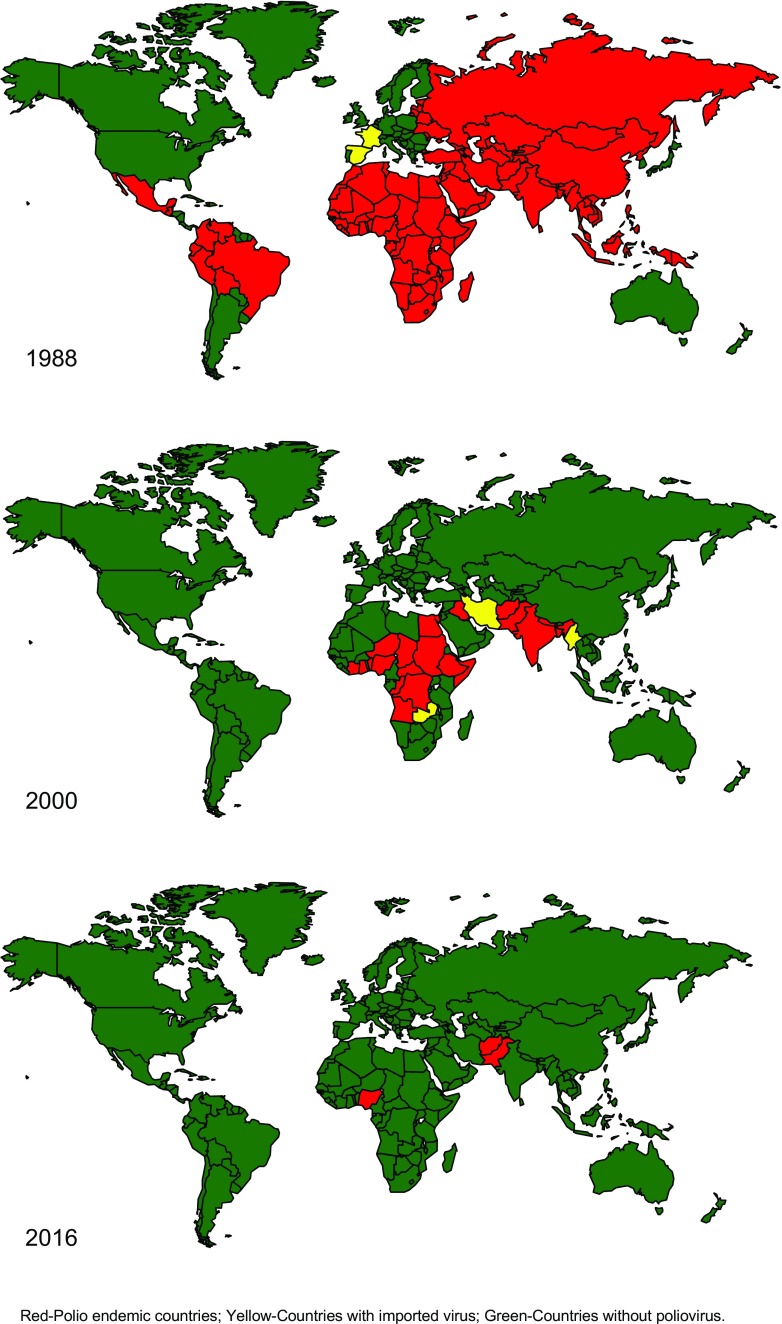
Polio-endemic countries, by year (1988, 2000, and 2016)

Substantial progress toward the eradication target has been accomplished since 1988 [1]. The eradication efforts can be divided into several distinct phases. The first phase was from the resolution of the World Health Assembly in 1988 to the respective target date, the end of the year 2000 [2] (Fig. 1). This period was marked by efforts to implement a global program, including strategy development, resource mobilization, field implementation, and rapid progress. The so called “low-hanging fruits” (*i.e*., countries with good health infrastructure or a specific focus on the goal) could implement the eradication strategies rapidly, and after only a few national vaccination campaigns were able to raise the immunity levels above the thresholds for herd immunity. As a result, these countries achieved eradication, and were removed from the list of polio-endemic countries. By the year 2000, the number of polio-endemic countries was 20 (compared with >125 in 1988), and the paralytic poliomyelitis cases had decreased by >99% (from >350,000 estimated cases in 1988 to 2849 reported cases in the year 2000) [3].

The second phase of the eradication efforts began in 2001, and ended in the early 2010s. This phase lasted for about a decade and was characterized by focusing increasingly on “difficult” areas [fewer countries, but major difficulties in accessing marginal populations (particularly in security compromised areas), and in general, suboptimal program performance in implementing the field activities, especially vaccination campaigns]. Because wild poliovirus circulation had already been suppressed in many countries, even in the then polio-endemic countries, the levels of immunity required to achieve interruption of transmission, now had to rely almost exclusively on vaccine-induced immunity, requiring frequent and massive mass vaccination campaigns. In spite the challenges, progress was made, and the number of endemic countries decreased from 20 in 2000 to 4 in 2010, but the number of cases largely stagnated between 483 and 1979 (Fig. 1) [4].

The third phase began in 2011 with the realization of polio eradication in India [5]. This achievement brought new energy, focus, political commitment, and finally, for the first time, adequate resources to the eradication initiative. Furthermore, real optimism was palpable, India had proved that the almost unsurmountable biological challenges (immunity threshold close to 100%) could be overcome, and with it the final proof of feasibility of eradication was provided. However, by now, endemic and epidemic transmission was limited to the three countries where eradication was most difficult to achieve. Pakistan (and to a lesser degree, Afghanistan) were delayed by access challenges and security concerns. Terrorist organizations targeted specifically polio health workers, and >100 health care workers lost their lives between 2012 and 2016 [6]. In Nigeria, the program had a period of about 2 y without detection of wild poliovirus type 1 (between mid-2014 and mid-2016), but in August 2016, wild poliovirus type 1 was detected again in Borno State, North eastern Nigeria, where an extremist group (Boko Haram) controls access, and does not allow vaccination [7]. Global eradication requires access to the communities, and therefore, unless access is achieved, the initiative will not be able to achieve the interruption of virus in the final reservoir in Nigeria. Ultimately, this third phase will end with global certification of wild poliovirus eradication.

Despite specific challenges in the different phases, the Global Polio Eradication Initiative (GPEI) is aggressively implementing the Polio Eradication and Endgame Strategic Plan 2013–2018, a comprehensive, long-term strategy that addresses what is needed to deliver a polio-free world [8]. A major objective, the removal of Sabin poliovirus type 2 from the oral poliovirus vaccine (OPV) was implemented in April 2016, following certification of wild type 2 poliovirus eradication. This globally synchronized effort represents the largest “recall” effort of a medicinal product ever. Over 150 countries prepared national plans, implemented the field activities and monitored this effort very closely [9]. At the same time, countries introduced bivalent OPV (bOPV), containing types 1 + 3 Sabin poliovirus, and where not done so previously, added at least one dose of inactivated poliovirus vaccine (IPV) into their routine immunization schedule to maintain an immunity base against poliovirus type 2 [10]. This effort was made possible because of the very close collaboration of GPEI partners, civil society organizations, countries and vaccine producers.

In April 2016, therefore, the GPEI has entered the post-OPV2 cessation era. Although the withdrawal planning placed a high priority on the identification and destruction of the remaining trivalent OPV (tOPV) vaccine stocks, it has become apparent that some tOPV vials were missed, and more importantly, there is also evidence that some of this vaccine continues to be used [11]. The emergence and possible circulation of vaccine-derived poliovirus type 2 is a growing concern in the presence of decreasing mucosal immunity against type 2. In addition, although adequate IPV supply for a single dose routine strategy was committed by industry, the challenges faced with rapidly expanding production led to lower deliveries than anticipated (currently <50% of initial commitments). The shortages are affecting more than 30 countries that could not introduce or could not be re-supplied with IPV. These countries are on a holding path to access IPV, currently anticipated for 2018–2019.

In India, the switch from tOPV to bOPV went smoothly, but here as in some other countries, some tOPV stocks were not identified for destruction [10, 11]. Further auditing has been conducted (as part of the National Immunization Days), and it appears that some, mostly private sector providers were missed during the information campaign about the recall. For IPV, very proactive thinking and action, has ensured that the country is implementing a routine IPV schedule that is both more immunogenic than the one full dose recommendation and is dose-sparing. India has been rapidly expanding a schedule of two fractional IPV (fIPV, 1/5 of a full dose) given intradermally at 6 and 14 wk of age. Two doses of fIPV induce significantly higher seroconversion rates and antibody titers than the one full-dose schedule [12, 13]. On the other side, the programmatic implementation requires training and enhanced supervision.

This report outlines the remaining challenges to eradication, and the programmatic needs. This is phase four of the eradication effort, from certification of wild poliovirus eradication to validation of absence of all polioviruses from populations, and a strong infrastructure in place to deal with the invariable emergencies (*i.e*., emergence, transmission or release of poliovirus) as well as to control polio outbreaks. All these elements are critical to secure polio eradication for perpetuity.

## The “Way Ahead”

The “rocky” road to eradication: On a global level, the program is planning the necessary follow on actions after eradication. Indigenous wild poliovirus type 2 was last detected in Northern India in 1999, and the world certified free of wild poliovirus type 2 in 2015 [14]. This allowed the removal of Sabin type 2 from the OPV vaccine [9]. Wild poliovirus type 3 was last detected in Nigeria in 2012 (approximately 4.5 y ago) [1]. At this point, only wild poliovirus type 1 continues to circulate in parts of three countries (Northeastern Nigeria, Eastern & Southern Afghanistan and Western Pakistan). Areas in all these countries with type 1 virus, and indeed where the reservoirs are likely to persist, are in difficult to access areas, usually due to security concerns. These reservoirs occasionally export the virus into polio-free areas in the same country or across borders. Consequently, the achievement of eradication is dependent on the program’s ability to interrupt virus transmission in these last reservoirs.

At the same time, the GPEI must address the “zoo” of vaccine-related polioviruses (*i.e*., Sabin, vaccine-derived, and both circulating VDPV [cVDPV], and immunodeficient-associated VDPV [iVDPV]) [15]. It will be a challenge to convince the global community that polio eradication has been achieved when individuals continue to be paralyzed by vaccine-related polioviruses. Furthermore, given the predictable genetic evolution (mutation and recombination) of polioviruses, a failure to address this “zoo” would inevitably lead to re-establishment of endemic and epidemic transmission, and negate the achievements of eradication [16].

To address the “zoo”, including the type 2 virus, the following areas-of-work are a current priority: 1) the expansion of surveillance to detect cases with signs and symptoms of immunodeficiency to determine whether any of these cases are excreting poliovirus; and 2) the completion of the development of effective antiviral agents to eliminate such viral replication and excretion [17–19]. Although progress has been made in both areas, sensitive surveillance for primary immunodeficiency disorders (PIDs) that covers middle- and low-income countries is yet to be achieved.

**Fig. 2:**
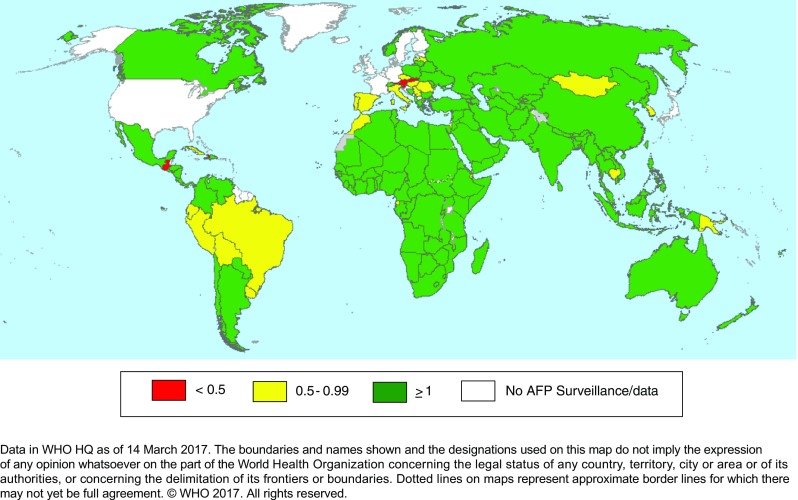
Non-polio acute flaccid paralysis (AFP), by 1 y period (February 2016 to January 2017) and country

Global certification (eradication surveillance quality and facility containment): Global certification of eradication requires, as a minimum, at least three years after the last detection of wild poliovirus globally in conditions of high quality surveillance [20]. The hierarchical review of evidence will rely on National Certification Committees (NCC), reporting to the Regional Certification Commissions (RCC), and the RCC in turn, will report to the Global Certification Commission (GCC) for final review and decision-making. The Regional certification processes were successfully concluded in the Region of the Americas in 1994 [21], in the Western Pacific Region in 2000 [22], in the European Region in 2004 [23], and in the South-East Asia Region in 2014 [24]. Two Regions, the African and Eastern Mediterranean Regions, are pending Regional certification, but in each of the Regions, the vast majority of countries have also been polio-free for many years and sometimes decades, and these countries have provided annual reports through the NCC to the respective RCC. Surveillance quality is a major consideration (Fig. 2). The GCC has once, in 2015, certified that one of three serotypes, wild type 2, has been eradicated [14].

Although these Regional certification processes have been successful, meaning that no Region “certified as polio-free” has ever again detected indigenous poliovirus circulation, the stakes will be invariably higher for global certification. The GCC is currently evaluating whether the “three-year rule” from last detection to certification remains valid, and whether the certification of wild poliovirus eradication should be followed by the validation (certification) of the absence of vaccine-related polioviruses from populations. Furthermore, the GCC has assumed oversight of the containment of polioviruses in laboratories and vaccine production sites. However, the prolonged and chronic poliovirus excretors could present a serious threat to eradication. These individuals could cause re-established renewed poliovirus transmission [15, 25, 26]. A single-drug regimen is available under an Investigational New Drug (IND) therapy for prolonged or chronic excretors. However, to prevent the development of resistance, a second drug is required [18].

**Fig. 3:**
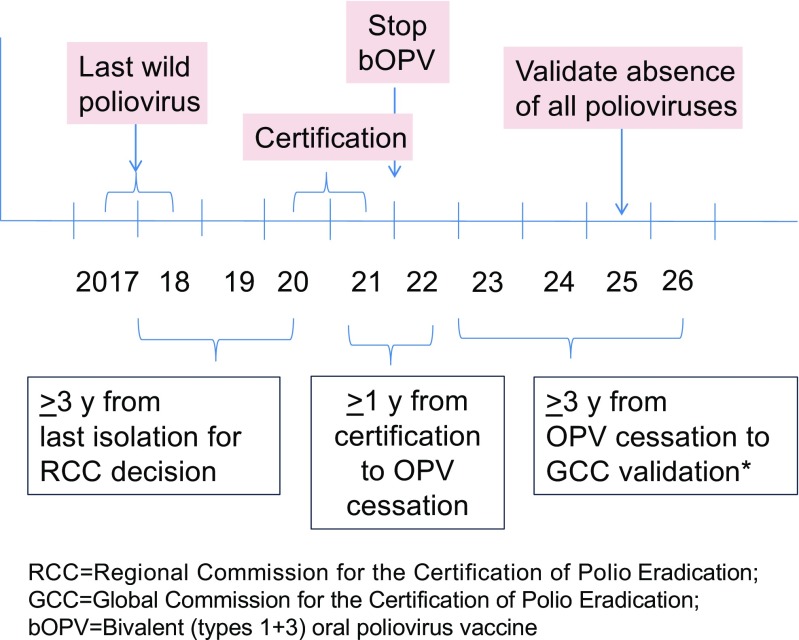
Anticipated polio eradication timeline

Cessation of all Sabin viruses in bOPV (types 1 and 3): As soon as possible after global certification of wild poliovirus eradication, while allowing sufficient time for appropriate planning, all Sabin viruses should be removed from communities [27]. The timeline for eradication is complex (Fig. 3). This means that the bOPV used primarily for polio prevention since the switch in April 2016 would no longer be available. Similar to the switch in 2016 [9], the complete removal of all Sabin strains (complete withdrawal of OPV), requires another globally synchronized effort. This would affect all bOPV-using countries (currently approximately 150 countries). The programmatic efforts would focus on careful country-level planning, implementation of “removal” (identification of supplies and destruction), and monitoring [9].

After the removal of OPV, the only vaccine available for polio prevention will be IPV. Substantial challenges still need to be addressed to ensure adequate production and supply of affordable IPV. In addition, funding support for IPV for the poorest countries (currently Gavi, the Vaccine Alliance, is providing this support to 71 Gavi eligible or recently graduated countries) should also be available. In the next 5–10 y, combination hexavalent vaccine containing IPV (DTwP-HepB-Hib-IPV) should become available and affordable for use in middle- and low-income countries. In India, the first such hexavalent vaccine has recently been licensed [28]. It is expected that other manufacturers will follow.

**Fig. 4:**
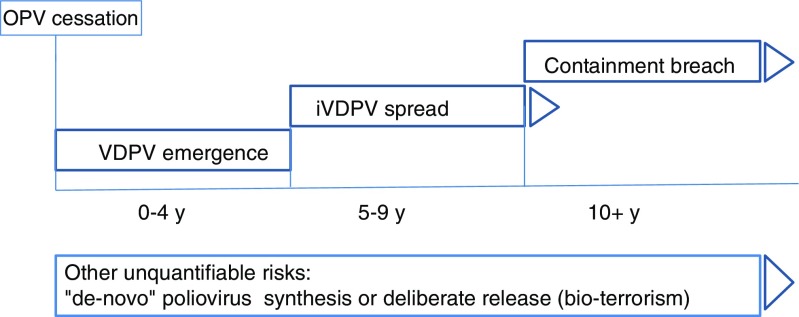
Predominant risks of poliovirus transmission into the general population following cessation of oral poliovirus vaccine (OPV).

Future vaccine options and immunization policy: Unlike smallpox [29], where vaccine production did not require smallpox virus, production of all current polio vaccines, whether live attenuated or inactivated, requires the growth of live polioviruses (Sabin for OPV, and Salk or Sabin for IPV). This entails that many laboratories and production facilities will need to continue to work with polioviruses for the foreseeable future. Also unlike smallpox, chronic infection, replication, and excretion has been demonstrated in individuals with PID. The longest documented excretor continues to excrete virus after ~30 y [30]. For these reasons, vaccination to induce an immunity base against poliovirus needs to continue. In addition to the risk of transmission of the virus from individuals with PIDs, the threat of containment breaches at facilities, the possibility of de-novo synthesis of virus, as well as the potential deliberate release, provide further justification for continuation of vaccination, even after certification of polio eradication and the removal of live attenuated poliovirus vaccines (Fig. 4).

Currently, there are ongoing discussions on what this immunity base would look like, and for how long the immunity induced by vaccination would be needed. The deliberations focus on a sero-conversion target of approximately 90% that a new routine immunization schedule should ideally achieve [31]. Two doses of IPV [whether full, or fractional (1/5 of a full dose)] could achieve this target (if given later in infancy and with a longer interval between doses) [32]. A new routine vaccination schedule that administers doses at 14 wk (with pentavalent vaccine) and at 9 mo (with measles vaccine) is being considered [33]. The Strategic Advisory Group of Experts (SAGE), the principle scientific oversight committee for vaccines and immunization, in April 2017 endorsed the 2-dose routine IPV schedule and a 10-y minimal duration of IPV use after OPV withdrawal, primarily to allow most chronic excretors in middle- and low-income countries to clear the infection or, unfortunately, expire.

Maintaining polio-essential functions: Similarly, discussions have started to define the polio-essential functions that will need to be sustained to maintain a polio-free world. A next Strategic Plan of Action (Post Certification Strategy) is therefore being developed to guide efforts for the period after global polio eradication has been certified by the GCC [34].

At the global level, four goals have been identified for the Post Certification Strategy to sustain a polio-free world (Fig. 4). The strategy addresses four goals: Goal one is to contain polio sources through implementing appropriate containment for poliovirus in facilities (laboratories and production sites). Containment, as one of the main pillars for a polio-free world, will rely on implementation of the WHO Global Action Plan to minimize poliovirus facility-associated risk after type-specific eradication of wild polioviruses and sequential cessation of oral poliovirus vaccine use– GAP-III [35]. Goal two of detecting and responding will address the surveillance and outbreak response needs for the future including how to address prolonged poliovirus excretors among individuals with PIDs. Goal three of protecting populations addresses the need to remove bOPV and maintain population immunity at a satisfactory level through routine immunization efforts. Finally, goal four of managing effectively and monitoring will address the coordination mechanisms needed to support the program.

Transition: WHO is in the process of assessing the programmatic, financial, and human-resource-related risks resulting from the winding-down and eventual discontinuation of the GPEI. It is also looking at efforts underway and planned to mitigate those risks while ensuring that essential polio-related functions are maintained. A report will be presented to WHO Member States in 2017. The GPEI has already started to ramp down; with both human and financial resources becoming increasingly scarce. Multiple surveys have demonstrated that the polio eradication infrastructure has been used to contribute to other health priorities (for *example* for routine immunization, new vaccines introductions, emergencies, outbreak response, mass campaign planning and implementation, *etc*.). In some WHO Regions, the GPEI funding constitutes more than 40% of the regional budget expenditures, and supports a large proportion of all immunization staff. To avoid negative effects to other health program, the ramp down must be carefully planned and implemented gradually. In the past, these programs could rely on GPEI resources (polio-funded staff, vehicles, and funding). In the 16 GPEI priority countries (that currently receive 95% of GPEI resources), transition plans are being developed, and in each country the priorities identified, and potential solutions ascertained to find adequate financial support to make up, when deemed necessary, for the loss of GPEI funding. The other health programs thus impacted will need to own the transition process, and actively find, when deemed necessary, innovative solutions and funding. A high level Transition Independent Monitoring Board (TIMB) has been constituted to monitor progress and provide independent advice.

“Looking into a crystal ball” – the future with vaccine product development: The development of poliovirus vaccines will focus increasingly on IPV and vaccines that do not require infectious processes for virus growth. IPV will increasingly be produced from Sabin strains, and further attenuated or genetically-modified strains. Ultimately, polio vaccines will be produced by non-infectious processes, such as Virus-Like Particles (VLPs), or packing-cell technology. Both of these can provide immunogenic non-infectious vaccines [36, 37]. However, although these vaccines are expected to prevent paralytic disease through humoral immunity following poliovirus exposure, their ability to induce mucosal immunity is limited, and may be the major limitation. Therefore, the efforts to develop mucosal adjuvants for these new generation vaccines is a high priority. The development of new oral poliovirus vaccine type 2 (nOPV2) is expected to respond to this critical need (induce mucosal immunity). Until then, the mOPV2 from stockpiles can be used.

## India

Maintain polio-free India: Once considered the most difficult place in the world to eliminate wild poliovirus, India achieved this success through a relentless focus on reaching and immunizing every last child with OPV, not once but repeatedly. The success against polio in India was an outcome of committed leadership, dedication of the health workers, tailored strategies, data driven planning through surveillance and research, rigorous monitoring, targeted communication and social mobilization efforts, strong partnerships and adequate funding. An internal accountability framework ensured that all strategies were implemented with the rigor that is required for disease elimination. India has maintained its polio-free status for more than 6 y, since the detection of the last case due to wild poliovirus in January 2011 in Howrah district (West Bengal). It continues to maintain high immunization coverage, especially in the hard-to-reach areas and among the most vulnerable populations, through regularly conducted special polio vaccination campaigns. Efforts to improve overall routine immunization coverage, including against polio, have been intensified in districts with suboptimal performance, as part of the national flagship program -“Mission Indradhanush” which is a health mission of the government of India to immunize all children under the age of 2 y against seven vaccine preventable diseases. Cross-border coordination to ensure vaccination of populations along the international borders with Nepal, Pakistan, Bangladesh and Bhutan has been continued.

Surveillance performance in India continues to surpass the globally recommended standards. As a supplement to the ongoing nation-wide AFP surveillance, India has expanded environmental surveillance to 8 states (35 sites) compared to 2 states (8 sites) in 2010. More than 1100 sewage samples collected annually are being tested for any wild, vaccine-derived or Sabin-like polioviruses annually in India. Going forward, environmental surveillance will play an increasingly important role in detecting polioviruses.

India switched from tOPV to bOPV on 25 April 2016 as a part of the globally synchronized withdrawal of the type 2 component of OPV and more than 21,000 cold chain points and health facilities were monitored as a part of the validation exercise following the switch. Accelerated environmental surveillance identified sewage samples in two states which tested positive for Sabin-like type 2 polioviruses, about four months after the switch. A detailed physical street-by-street survey undertaken in the districts where Sabin like type 2 polioviruses were detected and a follow-up nationwide search in more than 250,000 health facilities during the NID, have detected tOPV being used in a few centers, mostly small private sector providers [11]. Valuable lessons learned from the switch and the post-switch surveys will be extremely useful during the total withdrawal of all OPV, post global certification.

IPV was introduced in the EPI program in selected states in November 2015, with plans to expand to the other states by mid-2016. In the context of the global IPV shortage, and based on recommendations of the India Expert Advisory Group (IEAG), India moved, in a phased manner, to a two fractional-dose IPV schedule. By June 2017, all states/Union Territories in India have switched to the fractional-dose schedule. Optimization of the timing of the two-dose schedule will have to be considered based on evidence generated and programmatic needs as determined by the global epidemiology of polioviruses.

India national transition planning: Over the last two decades, critical support has been provided to the polio eradication initiative in India by the WHO National Polio Surveillance Project (NPSP), the UNICEF Social Mobilization network as well as the CORE group of non-governmental organizations (NGOs). While maintaining ongoing support for polio surveillance and immunization, NPSP/WHO India is getting increasingly involved with efforts to strengthen immunization systems and promoting health equity for achieving high immunization coverage. With wide spread credibility and acceptance on the ground, the NPSP/WHO India workforce is transitioning knowledge, lessons and assets to support priority health areas of the government such as Mission Indradhanush, measles elimination and rubella control, surveillance of other vaccine preventable diseases and the introduction of new and under-utilized vaccines (rotavirus vaccine, pneumococcal vaccine and the human papillomavirus vaccine). A transition plan to mainstream essential polio functions and a simultaneous transfer of polio assets to achieve broader gains in public health is being developed. In the context of a ramp-down and an ultimate discontinuation of GPEI funding after 2019, alternative sources of funding, including from the government of India, will be critical to maintain continuity of operations and provide critical support for polio and other program priorities.

## Discussion

This communication attempts to sketch the “way ahead” of the polio eradication initiative. Since the World Health Assembly resolution in 1988, dramatic progress toward eradication has been achieved. The most note-worthy is that 15 million people are walking today and are not paralyzed by poliovirus, primarily as a result of the eradication efforts. As emphasized, the first priority is to achieve eradication, and the next priority is to secure eradication for perpetuity. The complexities of ending the GPEI and securing eradication are vast and will require coordinated efforts for many years to come.

As the GPEI increasingly focuses on the remaining three polio-endemic countries, the success of the initiative is dependent on gaining safe access to reach all unreached and unvaccinated children and to conduct quality surveillance. While the current progress in South Asia (Pakistan and Afghanistan) gives cause for optimism, the humanitarian situation of the African Lake Chad basin continues to give cause for worry.

To secure eradication, a number of polio-essential functions must be maintained for the medium term (10–15 y), and will require human and financial resources. Because polio is very different from smallpox [29], continuing vaccination with IPV for a substantial period of time will be necessary. This will provide insurance in the case of virus spread to communities from facility containment breaches or from those individuals with chronic poliovirus infection. At a minimum, the public health community needs to minimize the paralytic burden arising from these breaches. Progress toward developing affordable hexavalent combination vaccines should make these available and affordable for middle- and low-income country use.

The transition of the GPEI infrastructure to other health priorities is another extremely complex enterprise. At this point, the public health community is just starting to realize the implications and risks associated with the rampdown of the GPEI infrastructure and resources. The price for not completing this planning process is high, and will likely be paid by children in lowest income settings.

In summary, polio eradication will continue to occupy another generation of public health professionals. Although the war on polio has already achieved a lot, but in the end only the final battle will matter. For polio eradication, the final battle will be the complete removal of all polioviruses from communities (finding ways to detect and clear infection among immunodeficient individuals) and ensuring that “contained” polioviruses remain in appropriate facility containment [35].

## References

[1] Morales M, Tangermann RH, Wassilak SG (2016). Progress toward polio eradication - Worldwide, 2015-2016. MMWR Morb Mortal Wkly Rep.

[2] World Health Assembly. Global eradication of poliomyelitis by the year 2000: resolution 41.28. Geneva. Switzerland: World Health Organization; 1988.

[3] Centers for Disease Control and Prevention (CDC). Progress toward global poliomyelitis eradication. MMWR Morb Mortal Wkly Rep. 2001;50:320-2, 331. 11465901

[4] Kew O (2012). Reaching the last one per cent: progress and challenges in global polio eradication. Curr Opin Virol.

[5] Centers for Disease Control and Prevention (CDC) (2011). Progress toward poliomyelitis eradication---India, January 2010--September 2011. MMWR Morb Mortal Wkly Rep.

[6] McGirk T. Polio’s surprising comeback: Taliban Assassins Target Pakistan's Polio Vaccinators. National Geographic, 3rd March 2015.

[7] Nnadi C, Damisa E, Esapa L (2017). Continued endemic wild poliovirus transmission in security-compromised areas - Nigeria, 2016. MMWR Morb Mortal Wkly Rep.

[8] Global Polio Eradication Initiative (2013). Polio Eradication & Endgame Strategic Plan 2013–2018.

[9] Hampton LM, Farrell M, Ramirez-Gonzalez A (2016). Immunization Systems Management Group of the Global Polio Eradication Initiative. Cessation of trivalent oral poliovirus vaccine and introduction of inactivated poliovirus vaccine - worldwide, 2016. MMWR Morb Mortal Wkly Rep.

[10] Garon J, Seib K, Orenstein WA (2016). Polio endgame: the global switch from tOPV to bOPV. Expert Rev Vaccines.

[11] Bahl S, Hampton LM, Bhatnagar P (2017). Notes from the field: detection of Sabin-like type 2 poliovirus from sewage after global cessation of trivalent oral poliovirus vaccine - Hyderabad and Ahmedabad, India, August-September 2016. MMWR Morb Mortal Wkly Rep.

[12] Anand A, Molodecky NA, Pallansch MA, Sutter RW (2017). Immunogenicity of poliovirus type 2 following two intradermal fractional doses of inactivated poliovirus vaccine: a novel dose sparing immunization schedule. Vaccine.

[13] Okayasu H, Sein C, Chang-Blanc D, et al. Intradermal administration of fractional doses of inactivated poliovirus vaccine: a dose sparing option for polio immunization. J Infect Dis. 2017;216:S161–7. 10.1093/infdis/jix038 PMC5853966 28838185

[14] Global Commission for the Certification of Poliomyelitis Eradication. 14th Meeting of the Global Commission for the Certification of Poliomyelitis Eradication (GCC) Bali, Indonesia, 20–21 2015: Summary of findings, decisions and recommendations. Geneva, Switzerland: World Health Organization; 2015. p. 1–11.

[15] Kew OM, Sutter RW, de Gourville EM, Dowdle WR, Pallansch MA (2005). Vaccine-derived polioviruses and the endgame strategy for global polio eradication. Ann Rev Microbiol.

[16] Sutter RO, Cochi K, Aylward S. Poliovirus vaccine - live. Vaccines, 6th ed. In: Plotkin SA, Orenstein WA, editors. Philadelphia: WB Saunders; 2008. p. 631–85.

[17] Collett MS, Hincks JR, Benschop K (2017). Antiviral activity of pocapavir in a randomized, blinded, placebo-controlled human oral poliovirus vaccine challenge model. J Infect Dis.

[18] McKinlay MA, Collett MS, Hincks JR (2014). Progress in the development of poliovirus antiviral agents and their essential role in reducing risks that threaten eradication. J Infect Dis.

[19] Sutter RW, Modlin JF, Zaffran M (2017). Completing polio eradication: the case for antiviral drugs. J Infect Dis.

[20] Dowdle WR, Birmingham ME (1997). The biologic principles of poliovirus eradication. J Infect Dis.

[21] Robbins FC, de Quadros CA (1997). Certification of the eradication of indigenous transmission of wild poliovirus in the Americas. J Infect Dis.

[22] Centers for Disease Control and Prevention (CDC). Certification of poliomyelitis eradication--Western Pacific Region, October 2000. MMWR Morb Mortal Wkly Rep. 2001;50:1–3. 11215787

[23] The WHO European Region declared free of polio (2002). Euro Surveill.

[24] Bahl S, Kumar R, Menabde N (2014). Polio-free certification and lessons learned--South-East Asia region, March 2014. MMWR Morb Mortal Wkly Rep.

[25] Kew OM, Sutter RW, Nottay BK (1998). Prolonged replication of a type 1 vaccine-derived poliovirus in an immunodeficient patient. J Clin Microbiol.

[26] Guo J, Bolivar-Wagers S, Srinivas N, Holubar M, Maldonado Y (2015). Immunodeficiency-related vaccine-derived poliovirus (iVDPV) cases: a systematic review and implications for polio eradication. Vaccine.

[27] Expanded Programme on Immunization. Report of the 1st Meeting of the Global Commission for the Certification of the Eradication of Poliomyelitis Geneva, Switzerland, 16-17 1995. Geneva, Switzerland: World Health Organization; 1995 (WHO/EPI/GEN/95.6).

[28] Panacea Biotec Ltd. Panacea Biotec introduces World’s first fully liquid hexavalent combination vaccine EasySix™ for Six Preventable Diseases. Available at: www.panacea-biotec.com/press_releases/PR29032017.pdf. Accessed on 19 September 2017.

[29] Breman JG, Henderson DA (2002). Diagnosis and management of smallpox. N Engl J Med.

[30] Dunn G, Klapsa D, Wilton T, Stone L, Minor PD, Martin J (2015). Twenty-eight years of poliovirus replication in an immunodeficient individual: impact on the global polio eradication initiative. PLoS Pathog.

[31] World Health Organization (2016). Meeting of the Strategic Advisory Group of Experts on immunization, October 2016 – conclusions and recommendations. Wkly Epidemiol Rec.

[32] Resik S, Tejeda A, Sutter RW (2013). Priming after a fractional dose of inactivated poliovirus vaccine. N Engl J Med.

[33] World Health Organization (2017). Meeting of the Strategic Advisory Group of Experts on immunization, April 2017 – conclusions and recommendations. Wkly Epidemiol Rec.

[34] Centers for Disease Control and Prevention. Polio Endgame and Legacy. Available at: https://www.cdc.gov/polio/plan/. Accessed on 19 April 2017.

[35] World Health Organization. WHO global action plan to minimize poliovirus facility-associated risk after type-specific eradication of wild polioviruses and sequential cessation of oral polio vaccine use – GAPIII. Geneva, Switzerland: World Health Organization; 2015 (WHO/POLIO/15.05).

[36] Adeyemi OO, Nicol C, Stonehouse NJ, Rowlands DJ. Increasing type 1 poliovirus capsid stability by thermal selection. J Virol. 2017;91. pii: e01586–16. 10.1128/JVI.01586-16 PMC5286869 27928008

[37] Stony Brook University. Stable packaging cell lines for the same and efficient manufacture of vaccines against polio and other viruses. Available at: http://www.stonybrook.edu/research/otlir/technologies/Therapeutics/R-8166.pdf. Accessed on 4 April 2017.

